# Antioxidant Activity and Chemical Characteristics of Supercritical CO_2_ and Water Extracts from Willow and Poplar

**DOI:** 10.3390/molecules26030545

**Published:** 2021-01-21

**Authors:** Mateusz Ostolski, Marek Adamczak, Bartosz Brzozowski, Wiesław Wiczkowski

**Affiliations:** 1 Department of Food Biotechnology, University of Warmia and Mazury, 10-718 Olsztyn, Poland; mateusz.ostolski@uwm.edu.pl (M.O.); bartosz.brzozowski@uwm.edu.pl (B.B.); 2 Institute of Animal Reproduction and Food Research, Polish Academy of Sciences, 10-748 Olsztyn, Poland; w.wiczkowski@pan.olsztyn.pl

**Keywords:** green extraction, supercritical carbon dioxide, plant extract, polyphenols, flavonoids, antioxidant activity, HPLC-MS/MS, *Salix purpurea*, *Salix viminalis*, *Populus nigra*

## Abstract

Renewable feedstock from perennial industrial crops, including those cultivated on marginal land in a short-rotation coppice system, could be an important contribution to the bioeconomy. The majority of data available on the topic are limited to the production of bioenergy from this type of biomass. According to the concept of bioeconomy, biomass-based bioproducts have priority over energy production. This paper characterizes the chemical composition and antioxidant activity of extracts from bark (b), wood (w) or a mixture of bark and wood (b + w) from *Salix purpurea*, *Salix viminalis* and *Populus nigra* obtained using supercritical carbon dioxide (scCO_2_), scCO_2_ and water (1%, *w/w*) or only water. Generally, a high concentration of polyphenols was obtained after extraction with scCO_2_ and water, while the lowest concentration was found in extracts obtained with scCO_2_. The highest concentration of polyphenols (*p* < 0.05) was obtained in an extract from *P. nigra* (b) (502.62 ± 9.86 mg GAE/g dry matter (d.m.)) after extraction with scCO_2_ and water, whereas the lowest polyphenol concentration was observed in an scCO_2_ extract from *S. purpurea* (b) (6.02 ± 0.13 mg GAE/g d.m.). The flavonoids were effectively separated by extraction with scCO_2_ (0.88–18.37 mg QE/g d.m.). A positive linear relationship between the antioxidant activity determined by DPPH and ABTS assays and the concentration of polyphenols was demonstrated, R^2^ = 0.8377 and R^2^ = 0.9568, respectively. It is most probable that the concentration of flavonoids, rather than the concentration of polyphenols, determines the chelating activity of Fe^2+^. The Fe^2+^-chelating activity of scCO_2_ extracts ranged from 75.11% (EC_50_ = 5.41 mg/cm^3^, *S. purpurea*, b + w) to 99.43% (EC_50_ = 0.85 mg/cm^3^, *P. nigra*, b + w). The lowest chelating activity was demonstrated by the extracts obtained with scCO_2_ and water (maximum 26.36%, *S. purpurea*, b + w). In extracts obtained with scCO_2_ and water, *p*-hydroxybenzoic acid (210–428 µg/g), *p*-coumaric acid (56–281 µg/g), saligenin (142–300 µg/g) and salicortin (16–164 µg/g) were the dominant polyphenols. All of these chemical compounds occurred mainly in the free form. The *S. purpurea*, *S. viminalis* and *P. nigra* biomass proved to be an attractive source of biologically active compounds for various possible applications in food, drugs or cosmetics. These compounds could be extracted using an environmentally friendly method with scCO_2_ and water as a co-solvent.

## 1. Introduction

Plant extracts are a valuable source of biologically active compounds, mainly polyphenols [1]. Polyphenols are abundantly occurring organic compounds that feature a phenol ring or rings substituted with at least two hydroxyl groups and a side chain [1]. The main classes of polyphenolic compounds include phenolic acids, flavonoids, anthocyanins, stilbenes and lignans [1,2]. The large diversity of the structure of polyphenolic compounds determines their diverse biological activity. In food, polyphenols are responsible for color, flavor, odor and stability. Meta-analyses prove that a diet rich in non-nutrient polyphenols prevents many diseases, including civilization diseases [3]. These compounds were granted Generally Recognized as Safe (GRAS) status as they have a positive effect on human health [4,5] due to, among others, their antioxidant [6], anti-bacterial [7], anti-fungal [8] and anti-inflammatory [9] properties. Polyphenols are used in drug, functional food, dietary supplement and cosmetic production [10,11]. They prevent cancer [12], mitigate type-2 diabetes and demonstrate preventive and therapeutic potential for Alzheimer’s disease [13,14]. The global polyphenol market size was valued at 1.28 billion USD in 2018 and is expected to reach an estimated CAGR of 7.2% from 2019 to 2025 [15].

Based on the Phenol-Explorer database [16], the total polyphenol content, expressed as a sum of the content of individual polyphenols determined chromatographically, in the 100 polyphenol-richest food products ranged from 6.6 mg per 100 g in carrots to over 15,000 mg per 100 g in cloves [17]. Polyphenols usually require extraction prior to their application. The composition and the biological activity of polyphenols depend on the substrate and extraction method. There is no universal, flexible or optimal method for extraction of biological compounds. The various structures of polyphenols and the variety of physical and chemical characteristics require knowledge of the available extraction methods. Conventional solvent extraction is commonly used (e.g., tea preparation) [18] and traditional solid–liquid extraction is applied on an industrial scale [19]. The recognized disadvantages of conventional methods have resulted in the development and application of modern sustainable and green extraction techniques, i.e., the natural deep eutectic solvent (NADES) technique, supercritical fluid extraction, ultrasound- or microwave-assisted extraction, pressurized liquid extraction, high-pressure extraction and pressurized hot water extraction, as alternatives to conventional extraction methods [20,21]. These techniques may be promising for the extraction of phenolic compounds due to their advantages over the conventional methods, which include reduced solvent use, decreased time and energy consumption, no solvent residues and higher recovery rates at lower operational costs [22].

Plant biomass consists of many compounds occurring in the free or bound form, featuring diverse structures and biological or physico-chemical properties. Their polarity and diversified thermal stability determine the selection of extraction techniques and conditions that play a key role in the quality of extracts [23]. Although free phenolic compounds are solvent-extractable, bound phenolic compounds (which are covalently bound to the plant matrix) cannot be extracted into water or organic solvents. Data published in this paper on the contents of phenolic acids in *S. purpurea*, *S. viminalis* and *P. nigra* extracts are limited to water or alcohol extracts. This paper presents original and unique data concerning the composition of free and bound compounds present in extract obtained with supercritical carbon dioxide (scCO_2_). This information is valuable because conjugated and bound phenolics could play an essential role in releasing antioxidants to the colon by gastrointestinal tract microbiota in humans and animals [24]. Ethanol, water and scCO_2_ are considered safe and are preferentially used to obtain extracts for application in food and pharmaceutical production [22]. High selectivity and efficiency may be obtained by extraction with supercritical fluids, and the selectivity may be modified by adding co-solvents [25]. The main sources of phenolic compounds are fruits and vegetables, although waste and by-products [26,27,28] and woody vascular plants [29,30,31] (especially bark) are also a rich source of biologically active polyphenols [32,33,34,35]. The application of supercritical fluid and utilization of renewable substrate from perennial industrial crops, including those grown on marginal land using a short-rotation coppice system presented in this publication, could be an important contribution to the bioeconomy and circular economy [36,37]. Information on this complex topic is scarce in the scientific literature, and the majority of data are limited to the production of bioenergy from this non-food lignocellulosic substrate [38].

Due to the significance of polyphenols in the development of food properties and understanding of human health, the structural properties, activity and effect of these compounds are important, not only for selecting the extraction procedure but also for the application of the obtained extracts. Normally, polyphenols are present in the form of esters, glycosides and polymers and, because of the presence of hydroxyl groups, these compounds undergo various enzymatic modifications [39,40]. Antioxidant activity of polyphenols, which is caused directly through the reaction with reactive oxygen species (ROS) or indirectly through the stimulation of natural processes that improve the cell resistance to oxidative stress, is essential for human health. Due to different substitutions in the structure, polyphenols can inactivate many free radicals and oxidants, including superoxide, hydroxyl, peroxy radicals, singlet oxygen, nitric oxide and peroxynitrite [41]. Polyphenols can also act as chelators of transition metal ions Fe^2+^, Fe^3+^ and Cu^2+^, which are involved in the conversion of hydrogen peroxide into hydroxyl radicals and stimulation of lipid peroxidation [42]. Fe^2+^-chelating activity of plant extracts is of great importance because it has been proposed that the transition metal ions contribute to the oxidative damage in neurodegenerative disorders such as Alzheimer’s and Parkinson’s diseases [43]. It was also demonstrated that phenolic extracts are able to disintegrate the outer membrane of *Salmonella* strains by chelating divalent cations from the outer membrane [44].

Extracts from poplar or willow are usually obtained using water extraction, and limited information is available about the production and properties of these extracts. Water extracts from the bark of *S. purpurea* are used as ingredients in drugs and dietary and slimming supplements. Ecofriendly methods for extracting antioxidant compounds, using cheap, safe and abundant water as a co-solvent are preferred because the application of extracts obtained using organic solvents in food is limited due to the extract toxicity and long extraction times. The extract is used to treat the symptoms of fever, headache, inflammation and mild rheumatic complaints [45]. *S. viminalis* leaf ethanol extract may help to improve the quality and effectiveness of treatment in oncological diseases and mitigate the negative effects of treatment with such drugs as 5-fluorouracyl [46]. *Escherichia coli* growth-inhibiting effects of ethanol and organic solvent fractionated *Robinia pseudoacacia* extracts were demonstrated, as well as the positive impact of the extract during plaque removal and the treatment of periodontal inflammation [47]. Ethanolic extract from *Populus nigra* flower buds displayed a strong anti-inflammatory, hepatoprotective effect which indicates the possibility of its use as an ingredient in anti-inflammatory drugs [48].

The purpose of this paper was to characterize the total polyphenol and flavonoid concentration (TPC, TFC), antioxidant activity and Fe^2+^-chelating activity of extracts from *S.purpurea*, *S.viminalis*, and *P. nigra* obtained using scCO_2_, scCO_2_ and water (co-solvent) or water and determine the chemical composition of selected scCO_2_ extracts. For the first time, free and conjugated phenolic compounds were analyzed in willow and poplar bark and/or wood extracts obtained using scCO_2_.

## 2. Results and Discussion

### 2.1. Concentration of Polyphenols and Flavonoids

Short-rotation woody crops, such as fast-growing species, i.e., *Populus* and *Salix*, are commonly cultivated to yield biomass for bioenergy production since these trees can typically be harvested year-round and continue growing year after year. In Poland, this crop could also be planted on marginal land which, in Poland, is estimated to cover 2,000,000 ha of agricultural land. Furthermore, the bio-based economy concept assumes that biomass should not only be used for energy purposes. The raffinate obtained after extraction could be further used as a substrate for energy production [49]. After the preliminary experiments, the extract with the highest TPC from the bark of *S. viminalis* and extracts from the bark, wood or a mixture of bark and wood of *S. purpurea* and *P. nigra* were selected for analyses (Table 1). The quality of the extracts was influenced by the quality of the raw material, including its treatment before extraction as well as the extraction technique and parameters applied. Losses of polyphenol components were observed after the drying of willow bark by air-drying or tray-drying at a temperature above 70 °C [50]. Generally, the highest concentration of polyphenols was obtained after extraction with the use of scCO_2_ and water (B), followed by maceration in water (C), while the lowest concentration was found for the extracts obtained with scCO_2_ (A) (Table 1). The highest concentration of polyphenols (*p* < 0.05) was found in the product obtained from the bark of *P. nigra* (502.62 ± 9.86 mg GAE/g d.m.) after extraction with scCO_2_ and water, while extraction with scCO_2_ or water resulted in only 3.44% and 14.74% of the highest concentration of polyphenols, respectively (Table 1).

The lowest concentration of polyphenols was found in the scCO_2_ extract from the bark of *S. purpurea* (6.02 ± 0.13 mg GAE/g d.m.) (Table 1). No differences were found in polyphenol content in extracts obtained from *S. purpurea* (b + w) after maceration or extraction with scCO_2_ (63.92 and 66.30 mg GAE/g d.m, respectively) (Table 1). A low concentration of polyphenols was also found in the scCO_2_ extracts obtained from the *S. viminalis* (b) and the *P. nigra* (w, b, b + w). A lower content of polyphenols (*p* < 0.05) was obtained in the extract from *S. purpurea* (w) after maceration than following extraction with scCO_2_.

Todaro et al. [51] obtained the highest polyphenol concentration determined by using Folin-Ciocalteu reagent, which was over 300 mg GEA/g d.m. after thermo-treatment of poplar wood at 220 °C. Similar concentrations of polyphenols were obtained after applying a water/ethanol mixture (30/70, *v/v*) for maceration or ultrasound-assisted extraction or accelerated solvent extraction. In leaves of *Salix* species or hybrids, polyphenol concentration after extraction with methanol and HCl (99/1, *v/v*) ranged from 18.19 to 84.71 mg GAE/g d.m [52]. These values were similar to those obtained in this study after maceration and extraction with scCO_2_ and were much lower compared to those following extraction with scCO_2_ and water (Table 1).

Flavonoids are a class of polyphenols featuring various activity and basic structures consisting of C_6_-C_3_-C_6_ rings with different substitutions. Due to the wide diversity of the structures and activities, numerous analyses have been conducted in this respect [31,34]. The highest concentration of flavonoids was reported in the extracts obtained after extraction with scCO_2_ (0.88–18.37 mg QE/g d.m.) (Table 1), which indicates the hydrophobic nature of this group of chemicals present in the analyzed raw materials. A significantly higher (*p* < 0.05) concentration of flavonoids in comparison with other extracts was obtained from *S. viminalis* (bark (b)), *S. purpurea* (wood (w) bark and wood (b + w)) and *P. nigra* (b, b + w) after extraction using scCO_2._ Depending on the raw material used, the concentration of flavonoids in the extracts obtained using scCO_2_ and water or only water ranged from 0.47 to 6.68 mg QE/g d.m and from 0.49 to 1.99 mg QE/g d.m, respectively (Table 1).

The properties of supercritical fluids, green solvents and the clean technique enable rapid mass transfer and enhanced penetration capacity of the sample matrix, resulting in quick and efficient extraction [53]. However, scCO_2_ is not the best solvent for the extraction of polar compounds, including polyphenols [54]. Extraction with carbon dioxide modified by water improved the effectiveness of polar polyphenol extraction. Water content is among the key aspects affecting the extraction effectiveness. Water is considered to be soluble at approximately 0.3% (*v/v*) in scCO_2_. The presence of water, however, may either improve or reduce the diffusion of scCO_2_ [55]. A co-solvent may modify the properties of the raw material matrix as well as the solvent, and it may cause the formation of hydrogen bonds as well as dipol–dipol interactions between the co-solvent and the extracted compounds [56].

Piantino et al. [57] applied scCO_2_ (60 °C, 40 MPa) to extract polyphenol compounds from *Baccharis dracunculifolia*, the yield of which was 2-fold higher than that of methanol or ethanol. Kukula-Koch et al. [58] assessed the effect of the extraction method on polyphenol content and antioxidant activity of *Berberis cretica* extract. After the application of scCO_2_ and the addition of ethanol, higher extraction efficiency of polyphenolic compounds, higher content of volatile compounds and 5 to 10-fold higher antioxidant potential were obtained than after using pure scCO_2_.

The properties of supercritical fluids can be modified by the selection of temperature and extraction pressure. There are few comprehensive data concerning the extraction of polyphenolic compounds from bark or wood from plants cultivated in a short-rotation coppice system. Kuś et al. [59] found that scCO_2_ (30 MPa, 60 °C) could be used for green, efficient and simultaneous extraction of both volatile/non-volatile, bioactive phytochemicals of poplar buds and precursors of poplar-type propolis. Soares et al. [60] used supercritical fluid extraction of black poplar seeds to obtain polyunsaturated fatty acid concentrates. The highest yield (7.7 g extract/100 g dried black poplar seeds) was obtained at 80 °C and 25 MPa. Scalia et al. [61] assessed the possibility of the extraction of flavonoids (apigenin and apigenin-7-glucoside) from *Matricaria chamomilla* using scCO_2_ or extraction in a Soxhlet extractor or maceration with ethanol. They found that after a 30 min extraction with scCO_2_, similar apigenin release efficiency was obtained as after a 6 h extraction in a Soxhlet apparatus and it was higher than after a 3 days maceration period. However, the liberation efficiency of high polar apigenin-7-glucoside was very low after the application of scCO_2_ and did not increase significantly after the addition of 5% (*v/v*) methanol. Willow is an important bioenergy crop since its biomass can be accumulated in a relatively short time, while willow bark demonstrates a medicinal effect similar to that exerted by acetylsalicylic acid. The raw material from several species of willow is of pharmaceutical importance. However, substantial and significant cultivar-dependent variation was observed in the chemical composition of extracts obtained after sonication and methanol treatment [62].

### 2.2. Antioxidant Activity of Extracts

The highest antioxidant activity was observed for the extracts obtained with a mixture of scCO_2_ and water. In this extraction variant, the highest DPPH radical scavenging activity (*p* < 0.05) was reported for *S. purpurea* (11.86–15.25 g Trolox/g d.m.) and *P. nigra* (7.06–10.53 g Trolox/g d.m.) (Table 2). The lowest antioxidant activity was observed for extracts obtained with pure scCO_2_ and, depending on the applied raw material, the antioxidant activity of extracts ranged from 0.03 (*P. nigra*, w) to 1.74 g Trolox/g d.m. (*S. purpurea,* b + w).

The ABTS cationic radical scavenging capacity of extracts was also the highest (*p* < 0.05) for the extracts obtained using a mixture of scCO_2_ and water (Table 2). The antioxidant activity of *S. purpurea* (b) extract obtained using scCO_2_ and water was almost 30-fold higher than for an extract from the same material obtained using scCO_2_ and 7-fold higher than for the water extract from *P. nigra* (w) (Table 2).

The correlation coefficient of the antioxidant activity of the extracts by DPPH and ABTS assays and TPC was R^2^ = 0.8377 and R^2^ = 0.9568, respectively. Kiselova et al. [63] assessed the antioxidant activity of water extracts from 23 Bulgarian plants. A positive linear relationship between antioxidant activity and TPC was demonstrated (r = 0.92). Similarly, Vamanu and Nita [64] found a positive correlation between TPC, TFC, anthocyanin and tocopherol concentration and the antioxidant activity of extract from Romanian wild mushroom *Boletus edulis*.

### 2.3. Fe^2+^-Chelating Activity of Extracts

Iron is a reactive metal catalyzing oxidative changes in proteins, lipids and other cellular components. Since metal ions can cause lipid peroxidation and produce free radicals and lipid peroxides, the activity of metal chelation indicates antioxidant and antiradical properties [65]. Iron chelation activity increased with increasing concentrations of extracts (Figure S1), and the highest chelation activity was found in the extracts obtained by using only scCO_2_. A positive relationship between polyphenol concentration and Fe^2+^-chelating activity was not confirmed. After applying the highest analyzed concentration of scCO_2_ extracts (10 mg/cm^3^), the Fe^2+^-chelation ranged from 75.11% (*S. purpurea*, b + w) to 99.43% (*P. nigra*, b + w). Extracts obtained by aqueous maceration also demonstrated high Fe^2+^-chelating activity, ranging from 53.69% (*S. purpurea*, w) to 91.28% (*P. nigra*, b + w). The lowest EC_50_ was found for the extract from *P. nigra* (b + w) obtained using scCO_2_ or by maceration (0.85 and 3.23 mg/cm^3^, respectively) (Table 3).

Surprisingly, the lowest chelation activity was demonstrated by the extracts obtained using scCO_2_ and water (maximum 26.36% for extract from *S. purpurea*, b + w) and the activity of these extracts was below 50% for the concentrations used (Table 3). The ability of polyphenols to chelate metal depends on the unique structure of these compounds and the number of hydroxyl groups. Only chemical compounds featuring a specific structure are responsible for Fe^2+^-chelation [66]. Strong iron-binding properties have been confirmed for compounds containing the “iron-binding motifs” identified in their structures, e.g., quercetin [67]. Moreover, the interaction between polyphenols and metal ions may depend on their concentration [68]. The ability of the extract to chelate Fe^2+^ may be influenced by the content of flavonoids, the highest concentrations of which were also found in the extracts obtained with the use of scCO_2_ (Table 1). This was confirmed by Sarikurkcu et al. [69], who evaluated the chelation activity of iron ions in extracts from *Clinopodium vulgare* L. subsp. *vulgare* L., obtained after extraction in a Soxhlet extractor using acetone, methanol or water. The highest ability to chelate iron ions was demonstrated by the acetone extract, in which the highest concentration of flavonoids was also found.

### 2.4. Composition Analysis by HPLC-MS/MS

The extracts demonstrating the highest concentration of polyphenolic compounds, obtained with scCO_2_ and water, were selected for the HPLC-MS/MS analysis (Table 1). The concentration of the following compounds was determined: salicin, salicortin, saligenin, catechin, quercetin and naringenin, and the following acids: ferulic acid, sinapic acid, *p*-coumaric acid, syringic acid, protocatechuic acid, hydroxybenzoic acid and caffeic acid (Table 4).

The data presented in this paper extend the limited information [70] on the chemical composition of extracts from biomass, obtained using various extraction techniques.

Salicin and its derivatives found in the bark and leaves of *Salix* and *Populus* genera are responsible for the pharmacological activity of extracts [37,54,71]. Salicin has been recognized to contain, among others, analgesic or anti-pyretic properties and is used in rheumatism treatment. Similar properties are shown by saligenin and salicortin, which is a very unstable glucoside. Depending on the raw material, the concentration of phenolic compounds was as follows: 6.11–11.05 µg of salicin/g of extract, 142.18–300.69 µg of saligenin/g of extract and 16.09–164.61 µg of salicortin/g of extract (Table 4). The highest concentration of salicins was recorded in the bark extract from *P. nigra* and *S. purpurea* and was 444.65 and 360.49 µg/g extract, respectively (Table 4).

In the bark of purple willow, the salicin and salicortin concentration ranged from 3.04% to 10.96% [71]. A high concentration of salicins was found in the bark of *S. acutifolia* (12.06%), but only 0.04% in the bark of *S. viminalis*. Depending on the raw material (clone), a significantly higher concentration of salicins in the bark of *S. purpurea* was found, ranging from 1 to 25 mg/g, although the HPLC technique with UV detectors and evaporative light scattering detector (ELSD) was used for this determination [72]. The variation of the qualitative and quantitative analyses of phenolic compounds is extensive and concerns the variation between and within species. Different sample preparation and analytical techniques are used, which also affect the quality of the results. The application of NMR and high mass accuracy LC-MS-MS techniques enables the identification of an analog of salicin (salicin-7-sulfate) for further investigation of the safety and efficacy of plant materials [73].

Among the analyzed products, the highest concentrations of flavone compounds: naringenin, catechin and quercitin (29.25, 28.24 and 5.05 µg/g of extract, respectively) were found in the extracts from the *P. nigra* bark. Hydroxybenzoic acid (210.45–428.88 µg/g extract) prevailed among the determined phenolic acids, whereas the lowest concentration was found for caffeic acid (1.46–17.12 µg/g extract). All of the chemical compounds determined by HPLC-MS/MS assay occurred mostly in the free form (Table 4).

## 3. Materials and Methods

### 3.1. Chemicals

The following chemical reagents were used in the present experiments: dimethyl sulfoxide (DMSO, ≥99%, Stanlab, Lublin, Poland), methanol (≥99%, Stanlab, Lublin, Poland), Folin-Ciocalteu reagent (AKTYN, Suchy Las, Poland), sodium carbonate (≥99%, Stanlab, Lublin, Poland), gallic acid (≥98%, Sigma Aldrich, Poznań, Poland), 2,2-diphenyl-1-picrylhydrazyl, (DPPH, Sigma Aldrich, Poznań, Poland), 6-hydroxy-2,5,7,8-tetramethylchromane-2-carboxylic acid (Trolox, ≥98%, Sigma Aldrich, Poznań, Poland), iron(II)sulfate (≥99%, Sigma Aldrich, Poznań, Poland), ferrosine (97%, Sigma Aldrich, Poznań, Poland), ethylenediaminetetraacetic acid (EDTA, ≥ 99%, Sigma Aldrich, Poznań, Poland), diethyl ether (≥98%, Sigma Aldrich, Poznań, Poland), aluminium chloride hexahydrate (≥99%, POCH), quercetin (≥95%, Sigma Aldrich), 2,2′-azino-bis(3-ethylbenzothiazoline-6-sulfonic acid) diammonium salt (ABTS, Sigma Aldrich, Poznań, Poland), potassium persulfate (≥99%, Sigma Aldrich, Poznań, Poland), acetonitrile (≥99.9%, Sigma Aldrich, Poznań, Poland), formic acid (≥98%, Sigma Aldrich, Poznań, Poland), hydrochloric acid (Fluka, Poznań, Poland) and sodium hydroxide (Sigma Aldrich, Poznań, Poland).

### 3.2. Extraction Conditions

The samples of wood (w), bark (b) or a mixture of bark and wood (b + w) from *Salix purpurea* L. (90.4–92.6%, *w/w* d.m.), *Salix viminalis* L. Ekotur variety (91.2–93.0%, *w/w* d.m.) and *Populus nigra* x *P. maximowiczii* Henry *cv*. Max-5 (92.2–93.1%, *w/w* d.m.) cultivated in a short-rotation coppice (SRC) system, harvested in February 2018, were obtained from the University of Warmia and Mazury experimental fields located in Northeastern Poland. The choice of harvest time was guided by the agronomic rationality of obtaining this kind of biomass and by the assumptions of the project within which this research was conducted. Biomass was collected in the period when this type of biomass is usually harvested for energy purposes, which ensures adequate growth, regrowth possibilities and appropriate humidity. Details of the bark, wood and a mixture of the bark and wood preparations and the extraction using scCO_2_ were presented by Stolarski et al. [49]. Extraction was performed in three variants using (A) scCO_2_, (B) scCO_2_ and 40% (*w*/*w*) of water in a substrate (scCO_2_ and water) and (C) water maceration (raw material/solvent ratio, 1/10 (*w*/*v*), 70 °C, 24 h). Supercritical extraction was performed as a part of the research project (see Acknowledgements). The extraction under optimal conditions was performed at 40 °C and 33 MPa using a NATEX pilot plant (Austria) in two 40 dm^3^ extractors. The extraction was performed with scCO_2_ and scCO_2_ and water from two 5 kg batches. The extraction time was 9 h, and the efficiency was approximately 200 kg CO_2_ per kg of substrate.

To prepare the working solution, the product obtained by scCO_2_ and scCO_2_ and water extraction (A and B) was dissolved in DMSO and stirred for 24 h (1400 rpm, 22 °C). To this solution, a 5% (*w/v*) aqueous DMSO solution was added and mixed for 1 h to obtain the final concentration of the extract of 10 mg/cm^3^. The samples were separated through a cellulose filter and used for analysis or were stored at 4 °C until analysis. Extracts obtained by aqueous maceration (C) were used directly for analysis or were stored at 4 °C until analyzed.

### 3.3. Determination of Total Polyphenol Concentration

The total polyphenol content (TPC) was measured according to a modified method described by Singleton and Rossi [74]. An amount of 0.5 cm^3^ of Folin-Ciocalteu phenol reagent and 0.4 cm^3^ of a 7.5% (*w/v*) aqueous sodium carbonate solution were added to 0.1 cm^3^ of extract. Samples were degassed by vigorous mixing (IKA MS3 basic). After a 30 min incubation in the dark, counted from the moment when the sodium carbonate solution was added, the absorbance was measured at λ = 756 nm (Beckman DU 650 Spectrophotometer, Fullerton, CA, USA). The results are expressed as gallic acid equivalent (mg GAE/g d.m. of extract).

### 3.4. Determination of Total Flavonoid Concentration

The total flavonoid content (TFC) was measured using the method described by Lamaison and Carnat [75]. An amount of 0.8 cm^3^ of a 2% (*w/v*) AlCl_3_ × 6H_2_O methanol solution was added to 0.4 cm^3^ of extract. After a 10 min incubation in the dark, counted from the moment when the complexing reagent (AlCl_3_ × 6H_2_O) was added, the absorbance was measured at λ = 430 nm (Beckman DU 650 Spectrophotometer, Fullerton, CA, USA). The results are presented as quercetin equivalent (QE) and expressed in mg QE/g d.m. of extract.

### 3.5. Antioxidant Capacity Assay (DPPH and ABTS test)

Radical scavenging activity was determined using a modified method described by Blois [76]. An amount of 1 cm^3^ of DPPH radical solution (0.05 mM) was added to 1 cm^3^ of extract solution. After 30 min incubation in the dark, counted from the moment when the DPPH was added, absorbance was measured at λ = 517 nm (Beckman DU 650 Spectrophotometer, Fullerton, CA, USA). The results are expressed as Trolox equivalent in g/g d.m. of extract.

The antioxidant activity of the extracts relative to the scavenging capacity of the ABTS cation radical was also determined according to the method of Re et al. [77]. The ABTS radical cation was obtained by the reaction of a 7 mM aqueous ABTS solution with a 2.4 mM potassium persulfate solution in the dark for 14 h at room temperature. This solution was diluted with methanol (1/49, *v/v*) before the analysis. An amount of 0.010 cm^3^ of the extract (1 mg/cm^3^) was added to 1 cm^3^ of diluted ABTS. The water extracts obtained in option C were lyophilized and dissolved in water to obtain the required concentration. After 30 min of incubation at room temperature, the absorbance was measured at λ = 734 nm (Beckman DU 650 Spectrophotometer, Fullerton, CA, USA). The efficiency of radical neutralization by the tested extracts is expressed as the percent of Trolox Equivalent Antioxidant Capacity (TEAC). The reference test was the antioxidant activity of Trolox (1 mg/cm^3^).

### 3.6. Fe^2+^-Chelating Activity Assay

The chelating activity of Fe^2+^ was measured according to the method modified by Singh and Rajini [78]. Briefly, a series of dilutions were prepared from a working solution of the extract (2, 4, 6, 8, 10 mg/cm^3^; the extracts obtained in option C were previously lyophilized and dissolved in water). Following this, 0.2 cm^3^ of diluted extract was mixed with 0.2 cm^3^ of a 1 mM FeSO_4_ solution, and the reaction was initiated by adding 0.4 cm^3^ of 0.5 mM ferrosine. After a 10 min incubation, absorbance was measured at λ = 562 nm (Beckman DU 650 Spectrophotometer). An aqueous solution of EDTA was used as the standard. Chelating activity was calculated using the following Equation (1):

Chelating activity = [(A_c_ − A_s_)/A_c_] × 100 (%), (1)

where A_c_ is the absorbance of the control sample and A_s_ is the absorbance in the presence of the extract.

The concentration of extracts required to chelate 50% of Fe^2+^ (EC_50_) was calculated from a linear regression analysis.

### 3.7. Determination of Phenolic Acids, Flavonoids and Salicylic Compounds by HPLC-MS/MS

The profile and content of phenolic acids, flavonoids and salicylic compounds were determined according to the method modified of Płatosz et al. [79]. The samples were extracted with 1 cm^3^ of a mixture of methanol/water/formic acid (80/19.9/0.1, *v/v/v*) by stirring overnight at 10 °C. The solutions were centrifuged (13,200× *g*, 20 min, 4 °C), and the precipitants were re-extracted under the above conditions. The supernatants obtained were combined into a 2 cm^3^ volumetric flask. Next, phenolic acids and flavonoids (free and those released from soluble esters and soluble glycosides) were isolated from the extracts according to the following procedure. In the first step, the acidity of the extract solution was adjusted to pH 2 using 6 M HCl, and free forms of compounds (F) were extracted three times with 1 cm^3^ of diethylether using vortexing (60 s) and sonication (60 s) (VC 750, Sonics & Materials Inc., Newtown, CT, USA). After centrifugation (5000× *g*, 5 min, 4 °C), the ether extract was collected and evaporated to dryness under nitrogen at 35 °C. In the second step, 1 cm^3^ of 4 M NaOH was added to the extract remaining after the first step, and the mixture was placed in a nitrogen atmosphere and hydrolyzed for 4 h at room temperature. After acidification to pH 2 using 6 M HCl, compounds liberated from soluble esters (E) were extracted three times with 1 cm^3^ of diethylether using vortexing (60 s) and sonication (60 s). After centrifugation (5000× *g*, 5 min, 4 °C), the ether extract was collected and evaporated to dryness under nitrogen at 35 °C. In the third step, 0.2 cm^3^ of 6 M HCl was added to the extract remaining after the second step, and the mixture was hydrolyzed for 1 h at 100 °C. After the hydrolysis, the mixture pH was adjusted to pH 2 using 8 M NaOH, and compounds released from soluble glycosides (G) were extracted three times with 1 cm^3^ of diethylether using vortexing (60 s) and sonication (60 s). After centrifugation (5000× *g*, 5 min, 4 °C), the ether extract was collected and evaporated to dryness under nitrogen at 35 °C. All samples, extracts and samples with dry residue obtained after ether evaporation (which were then dissolved in 0.1 cm^3^ of 80% methanol) were centrifuged (13,200× *g*, 20 min, 4 °C) and injected to HPLC-MS/MS for analysis. Aliquots (2 µL) of extracts were injected into a HPLC system (LC-200, Eksigent, Dublin, CA, USA) equipped with a dual-channel pump, column oven, autosampler (set at 4 °C) and a system controller link to the Analyst 1.5.1 system. Chromatographic separations were conducted with a HALO C18 column (2.7 µm particles, 0.5 × 50 mm, Eksigent, Dublin, CA, USA) at 45 °C at a flow rate of 15 µL/min. The eluting solvents were A (water/formic acid, 99.05/0.95, *v/v*) and B (acetonitrile/formic acid, 99.05/0.95, *v/v*). The gradient was used as follows: 5% B for 0.1 min, 5%-90% B for 1.9 min, 90% B for 0.5 min, 5–90% B for 0.2 min and 5% B for 0.3 min. For HPLC-MS/MS analysis, a QTRAP 5500 ion trap mass spectrometer (AB SCIEX, Foster City, CA, USA) was connected to the Eksigent LC200 via an ESI interface. Optimal ESI-MS/MS conditions, including nitrogen curtain gas, collision gas, ion spray source voltage, temperature, nebulizer gas and turbo gas, were as follows: 25 dm^3^/min, 9 dm^3^/min, −4500 V, 350 °C, 35 dm^3^/min and 30 dm^3^/min, respectively. Qualitative and quantitative analyses (performed in triplicate) were carried out using Analyst Software (AB SCIEX, Canada) with the Multiple Reaction Monitoring (MRM), based on an analysis of selected external standards obtained from Sigma Chemical Co. (St. Louis, MO, USA) (Supplementary Data, Table S1). The calibration curves for this study have an R^2^ range of 0.9927–0.9997. The analyte detection parameters were LOD = 0.01–0.31 µg/cm^3^ and LOQ = 0.04–1.00 µg/cm^3^.

### 3.8. Statistical Analysis

All analyses were carried out in triplicate. The results were expressed as mean values ± standard deviation (SD). The same letter indicates results which do not statistically differ within the parameter under consideration (ANOVA Bonferroni test, *p* < 0.05). All analyses were performed using the STATISTICA 13.3 package (TIBCO Software Inc. 2017).

## 4. Conclusions

The current strong interest in the extraction of biologically active chemical substances, including polyphenolic compounds, is related to the extraction with the use of supercritical fluids. Carbon dioxide in a supercritical state is a safe, environmentally friendly green solvent. The extraction method and its parameters affect the quantity and quality of the biologically active compounds. It has been demonstrated that the amount of polyphenols extracted from *S. purpurea*, S. *viminalis* and *P. nigra* increases when water is used as a co-solvent with scCO_2_. The application of a short-rotation coppice system in the cultivation of perennial industrial crops provides a valuable source of polyphenolic compounds. The biomass can be used in biorefineries because of its energy value and valuable biologically active compounds. The extracts from the analyzed biomass can be considered important sources of natural antioxidants due to their high concentrations of polyphenols and flavonoids occurring mainly in the free form and their high antioxidant activity. At the same time, attention should be paid to the variability of this biological material and the influence of various agrotechnical parameters on its quality. The *S. purpurea*, *S. viminalis* and *P. nigra* biomass proved to be an attractive source of biologically active compounds for various applications in the food, drug and cosmetics industries, and it could be separated using an environmentally friendly method with scCO_2_ and water as a co-solvent. Further characteristics of the extract properties are required as well as their fractionation and purification.

## Figures and Tables

**Table 1:** Total polyphenol (TPC) and flavonoid (TFC) content in extracts obtained using: scCO_2_, scCO_2_ and water or water.

Scheme	TPC (mg GAE/g d.m.) *			TFC (mg QE/g d.m.) *		
	scCO_2_	scCO_2_ and Water	Water	scCO_2_	scCO_2_ and Water	Water
*S. viminalis* (b)	10.38 ± 0.13 ^m,l^	220.25 ± 6.96 ^g^	132.38 ± 0.19 ^h^	9.50 ± 0.03 ^c^	0.47 ± 0.07 ^k^	1.03 ± 0.01 ^k,j,i^
*S. purpurea* (b)	6.02 ± 0.13 ^m^	377.83 ± 5.32 ^b^	59.69 ± 0.90 ^j^	2.07 ± 0.02 ^g^	3.19 ± 0.08 ^f^	1.99 ± 0.02 ^g^
*S. purpurea* (w)	55.64 ± 0.72 ^j^	319.95 ± 3.12 ^e^	41.45 ± 0.26 ^k^	8.43 ± 0.18 ^d^	1.83 ± 0.06 ^h,g^	0.84 ± 0.02 ^k,j^
*S. purpurea* (b + w)	66.30 ± 0.90 ^j,i^	260.71 ± 1.15 ^f^	63.92 ± 0.60 ^j,i^	18.37 ± 1.44 ^a^	6.68 ± 0.11 ^e^	1.97 ± 0.01 ^h,g^
*P. nigra* (b)	17.30 ± 0.94 ^l^	502.62 ± 9.86 ^a^	74.07 ± 1.26 ^i^	10.28 ± 0.13 ^b^	0.95 ± 0.01 ^k,j,i^	1.12 ± 0.01 ^k,j,i^
*P. nigra* (w)	7.00 ± 0.11 ^m,l^	359.02 ± 5.42 ^c^	32.46 ± 0.49 ^k^	0.88 ± 0.04 ^k,j,i^	1.85 ± 0.03 ^h,g^	0.49 ± 0.02 ^k^
*P. nigra* (b + w)	16.6 ± 0.67 ^m,l^	335.45 ± 0.76 ^d^	72.14 ± 0.58 ^d^	3.33 ± 0.03 ^f^	1.33 ± 0.01 ^j,i,h^	1.52 ± 0.04 ^i,h,g^

* Mean values of three different determinations followed by standard deviation are presented; b—bark, w—wood, b + w—a mixture of bark and wood. The different letters (^a–m^) in the same group indicate significant differences between the samples (*p* < 0.05).

**Table 2:** Antioxidant activity of the extracts obtained using: scCO_2_, scCO_2_ and water or water, determined by the DPPH and ABTS assays.

Substrate	DPPH (g Trolox/g d.m.) *			ABTS (%) *		
	scCO_2_	scCO_2_ and Water	Water	scCO_2_	scCO_2_ and Water	Water
*S. viminalis* (b)	0.09 ± 0.00 ^n^	4.41 ± 0.02 ^f^	4.67 ± 0.02 ^f^	1.89 ± 0.03 ^j,i^	42.01 ± 1.39 ^d^	32.25 ± 0.25 ^e^
*S. purpurea* (b)	0.07 ± 0.00 ^n^	15.25 ± 0.04 ^a^	2.20 ± 0.02 ^j^	2.58 ± 0.26 ^j,i^	75.77 ± 1.28 ^b^	21.36 ± 1.34 ^f^
*S. purpurea* (w)	0.25 ± 0.00 ^n^	11.86 ± 0.02 ^b^	1.10 ± 0.01 ^l^	1.50 ± 0.35 ^j,i^	65.33 ± 1.41 ^c^	11.34 ± 0.14 ^h,g^
*S. purpurea* (b + w)	1.74 ± 0.05 ^k^	15.25 ± 0.01 ^a^	3.88 ± 0.06 ^g^	1.93 ± 0.35 ^j,i^	43.73 ± 1.04 ^d^	16.07 ± 0.52 ^g,f^
*P. nigra* (b)	0.74 ± 0.02 ^m,k^	10.03 ± 0.02 ^d^	2.66 ± 0.02 ^i^	4.70 ± 0.70 ^j,i,h^	84.19 ± 0.24 ^a^	20.75 ± 1.22 ^f^
*P. nigra* (w)	0.03 ± 0.00 ^n^	10.53 ± 0.01 ^c^	1.08 ± 0.02 ^l^	1.43 ± 0.09 ^j^	64.81 ± 0.91 ^c^	9.17 ± 0.57 ^i,h,g^
*P. nigra* (b + w)	0.10 ± 0.00 ^n^	7.06 ± 0.07 ^e^	3.15 ± 0.08 ^h^	3.76 ± 0.33 ^j,i,h^	73.60 ± 0.49 ^b^	15.33 ± 0.61 ^g,f^

* Mean values of three different determinations followed by standard deviation are presented; b—bark, w—wood, b + w—a mixture of bark and wood. The different letters (^a–n^) in the same group indicate significant differences between the samples (*p* < 0.05).

**Table 3:** The concentration of extracts obtained using: scCO_2_, scCO_2_ and water or water, required to chelate 50% of Fe^2+^ (EC_50_).

Substrate	EC_50_ (mg/cm^3^)		
	scCO_2_	scCO_2_ and Water	Water
*S. viminalis* (b)	5.76	n.d.	5.14
*S. purpurea* (b)	3.32	n.d.	5.52
*S. purpurea* (w)	2.41	n.d.	7.61
*S. purpurea* (b + w)	5.41	n.d.	5.99
*P. nigra* (b)	4.84	n.d.	7.24
*P. nigra* (w)	1.93	n.d.	6.49
*P. nigra* (b + w)	0.85	n.d.	3.23

n.d.—no data, activity below 50% in the range of concentrations used.

**Table 4:** Influence of substrate on salicylic compounds, flavonoids and phenolic acids concentration in extracts obtained with scCO_2_ and water.

Compound	*S. viminalis* (b)	*S. purpurea* (b)	*S. purpurea* (w)	*S. purpurea* (b + w)	*P. nigra* (b)	*P. nigra* (w)	*P. nigra* (b + w)
Salicylic compounds (µg/g extract) *							
Salicin	9.32 ± 0.01	11.05 ± 0.01	10.95 ± 0.01	6.57 ± 0.01	10.68 ± 0.11	6.11 ± 0.00	10.28 ± 0.06
Saligenin	203.03 ± 0.96	184.83 ± 0.96	142.32 ± 0.02	181.52 ± 0.96	295.17 ± 0.96	142.18 ± 0.17	300.69 ± 0.96
Salicortin	111.56 ± 0.11	164.61 ± 0.65	81.00 ± 0.06	16.09 ± 0.65	138.80 ± 1.30	63.30 ± 0.11	39.58 ± 0.13
Total content	323.91	360.49	234.27	204.17	444.65	211.59	350.55
Flavonoids (µg/g extract) *							
Catechin	13.16 ± 0.07	15.38 ± 0.04	10.40 ± 0.04	5.24 ± 0.00	28.24 ± 0.08	20.32 ± 0.10	4.49 ± 0.02
F/E/G	11.73/0.50/0.93	14.34/0.27/0.77	9.95/0.17/0.27	4.79/0.19/0.26	26.83/0.56/0.85	19.71/0.41/0.20	3.60/0.28/0.60
Quercetin	1.67 ± 0.00	1.71 ± 0.00	1.36 ± 0.00	2.03 ± 0.02	5.05 ± 0.01	2.65 ± 0.02	1.29 ± 0.00
F/E/G	1.47/0.12/0.08	1.54/0.09/0.07	1.26/0.04/0.07	1.82/0.08/0.13	4.91/0.06/0.07	2.57/0.04/0.04	1.17/0.06/0.06
Naringenin	11.59 ± 0.03	15.19 ± 0.03	3.94 ± 0.04	8.56 ± 0.02	29.25 ± 0.03	8.79 ± 0.03	14.46 ± 0.03
F/E/G	10.82/0.29/0.48	14.78/0.27/0.15	3.39/0.40/0.15	8.23/0.28/0.04	28.63/0.27/0.35	7.84/0.43/0.52	14.29/0.04/0.13
Total content	26.42	32.28	15.70	15.84	62.54	31.76	20.23
Phenolic acids (µg/g extract) *							
Ferulic acid	41.88 ± 0.03	14.68 ± 0.03	10.34 ± 0.06	23.42 ± 0.12	26.87 ± 0.06	7.84 ± 0.12	14.77 ± 0.03
F/E/G	41.50/0.23/0.14	14.36/0.13/0.19	10.22/0.11/0.01	23.02/0.26/0.14	26.39/0.39/0.09	7.61/0.19/0.05	14.42/0.22/0.12
Sinapic acid	19.30 ± 0.03	13.41 ± 0.04	11.50 ± 0.03	3.43 ± 0.00	14.24 ± 0.03	19.62 ± 0.09	17.77 ± 0.04
F/E/G	18.66/0.52/0.12	12.69/0.34/0.37	11.00/0.31/0.19	2.88/0.36/0.19	13.67/0.35/0.23	19.40/0.13/0.10	17.37/0.29/0.11
*p*-Coumaric acid	263.18 ± 0.61	128.82 ± 0.61	93.98 ± 0.09	82.51 ± 0.14	281.35 ± 0.60	56.03 ± 0.07	106.09 ± 0.59
F/E/G	257.83/4.20/1.16	124.80/2.88/1.14	92.19/1.68/0.11	79.03/2.83/0.65	278.40/2.00/0.95	54.65/1.35/0.03	105.26/0.77/0.06
Syringic acid	72.35 ± 0.34	39.98 ± 0.13	75.92 ± 0.33	33.80 ± 0.11	76.77 ± 0.32	76.11 ± 0.52	60.38 ± 0.10
F/E/G	46.20/9.77/16.38	20.30/7.70/11.99	55.65/10.53/9.74	16.37/6.86/10.58	53.40/10.97/12.41	59.40/6.89/9.83	42.63/9.95/7.80
Protocatechuic acid	36.79 ± 0.06	27.54 ± 0.06	14.77 ± 0.09	11.92 ± 0.02	19.45 ± 0.02	10.16 ± 0.04	18.34 ± 0.06
F/E/G	28.22/2.01/6.56	14.07/2.11/11.36	5.74/2.03/7.00	7.24/2.31/2.36	15.77/2.19/1.49	3.56/1.45/5.16	5.65/2.79/9.90
*p*-Hydroxybenzoic acid	422.79 ± 0.30	255.71 ± 0.45	210.45 ± 0.46	384.63 ± 0.46	428.88 ± 0.45	269.33 ± 0.43	330.50 ± 0.46
F/E/G	389.73/19.97/13.09	230.32/13.11/12.29	191.80/9.74/8.91	356.08/17.83/10.72	381.96/25.19/21.73	246.83/8.95/13.55	309.20/9.58/11.72
Caffeic acid	11.57 ± 0.04	2.90 ± 0.00	1.46 ± 0.01	3.94 ± 0.01	17.12 ± 0.07	5.71 ± 0.01	10.54 ± 0.04
F/E/G	11.17/0.28/0.12	2.58/0.18/0.14	1.30/0.08/0.07	3.73/0.13/0.08	15.64/1.39/0.09	5.54/0.09/0.08	10.05/0.36/0.13
Total content	867.86	483.03	418.42	543.63	864.68	444.80	558.38
Total phenolic concentration	1218.19	875.79	668.39	763.64	1371.87	688.15	929.16

* Mean values of three different determinations followed by standard deviation are presented; b—bark, w—wood, b + w—a mixture of bark and wood. F/E/G—polyphenols in free, ester or glycoside form.

## Data Availability

The data presented in this study are available in supplementary material.

## Supplementary Materials

The following are available online. Table S1: Selected qualitative and quantitative analysis data. Figure S1: Fe^2+^-chelating activity at various concentrations of plant extracts obtained using scCO_2_, scCO_2_ and water or water.

## Sample Availability

Samples of the compounds are not available from the authors.
