# Supplementary material for: Antioxidant Activity and Chemical Characteristics of Supercritical CO2 and Water Extracts from Willow and Poplar
Source: Molecules. 2021 Jan 21;26(3):545. doi: 10.3390/molecules26030545 (PMC7866000; doi:10.3390/molecules26030545)
Supplement: Supplementary file 1 [file molecules-26-00545-s001.pdf]

Antioxidant Activity and Chemical Characteristics of Supercritical CO_2 and Water Extracts from Willow and Poplar

SUPPLEMENTAL MATERIAL

Mateusz Ostolski [1], Marek Adamczak [1,*], Bartosz Brzozowski [1] and Wiesław Wiczkowski [2]

[1]Department of Food Biotechnology, University of Warmia and Mazury in Olsztyn, Poland; mateusz.ostolski@uwm.edu.pl (M.O.); bartosz.brzozowski@uwm.edu.pl (B.B.)
[2]Institute of Animal Reproduction and Food Research, Polish Academy of Sciences; w.wiczkowski@pan.olsztyn.pl (W.W.).
*Correspondence: marek.adamczak@uwm.edu.pl; Tel.: +48-89523-38-38 (M.A.)

Table S1. Selected qualitative and quantitative analysis data.

Compound	Precursor ion (m/z)	Daughter ion (m/z)	Declustering potential (V)	Collision energy (V)	Collision cell exit potential (V)	Retention time (min)	Calibration curve equatation	Coefficient of determination, R^2
Salicylic compounds								
Salicin	285	211	-100	-27	-13	1.27	y = 155594x + 295153	0.9953
		123	-100	-20	-10			
Saligenin	123	121	-65	-16	-11	1.18	y = 218332x + 147009	0.9967
		65	-65	-39	-8			
Salicortin	421	155	-90	-30	-15	1.42	y = 173303x + 157583	0.9979
		123	-90	-20	-15			
Flavonoids								
Catechin	289	245	-100	-22	-16	1.22	$y = 3\times10^6x - 49118$	0.9995
		109	-100	-40	-15			
Quercetin	301	151	-115	-25	-15	1.69	$y = 3\times10^6x + 898845$	0.9989
		171	-115	-20	-13			
Naringenin	271	151	-100	-26	-12	1.52	$y = 2\times10^8x + 944156$	0.9973
		119	-100	-38	-12			
Phenolic acids								
Ferulic acid	193	134	-140	-10	-24	1.51	$y = 2\times10^6x + 403883$	0.9978
		178	-140	-17	-11			
Sinapic acid	223	208	-100	-20	-20	1.71	$y = 1\times10^7x - 262192$	0.9979
		164	-100	-21	-12			
p-Coumaric acid	163	119	-90	-20	-12	1.49	$y = 1\times10^7x + 328132$	0.9996
		93	-90	-40	-14			
Syringic acid	197	182	-100	-10	-15	1.37	y = 80829x + 434.01	0.9997
		153	-100	-15	-12			
Protocatechuic acid	153	109	-100	-10	-15	1.45	$y = 1\times10^6x + 149056$	0.9987
		91	-100	-20	-15			
p-Hydroxybenzoic acid	137	93	-100	-20	-10	1.67	$y = 1\times10^6x + 256655$	0.9972
		65	-100	-30	-12			
Caffeic acid	179	135	-90	-25	-15	1.33	$y = 6\times10^6x + 3\times10^6$	0.9927
		107	-90	-30	-15			

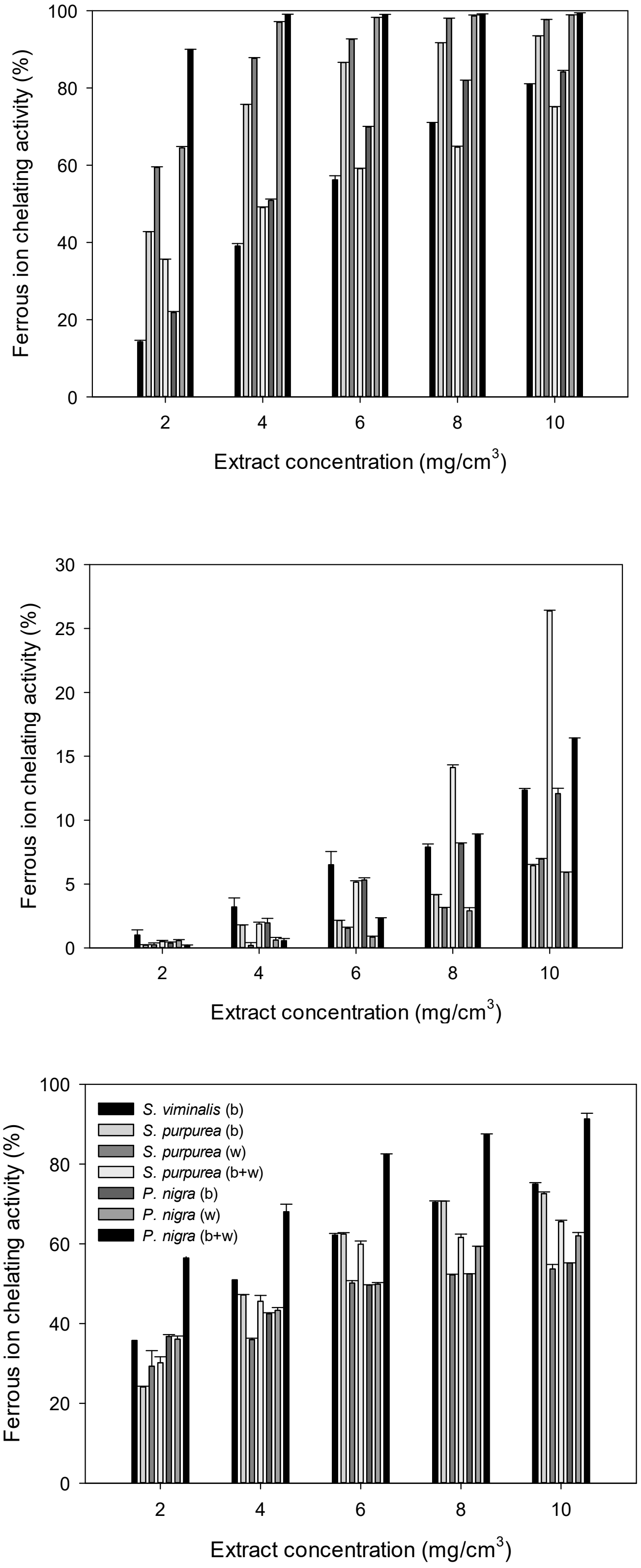

Figure S1. Fe^{2+}-chelating activity at various concentrations of plant extracts obtained using (A) $scCO_2$, (B) $scCO_2$ and water or (C) water. The legend for all figures is presented in Figure C. Mean values of three different determinations followed by standard deviation are presented.
